# Cytogenetic Analysis of the Bimodal Karyotype of the Common European Adder, *Vipera berus* (Viperidae)

**DOI:** 10.3390/ani12243563

**Published:** 2022-12-16

**Authors:** Victor Spangenberg, Ilya Redekop, Sergey A. Simanovsky, Oxana Kolomiets

**Affiliations:** 1 Vavilov Institute of General Genetics, RAS, Moscow 119991, Russia; 2 Severtsov Institute of Ecology and Evolution, RAS, Moscow 119071, Russia; 3 Moscow Region State Pedagogical University, Mytischi 141014, Russia

**Keywords:** prophase I of meiosis, synaptonemal complex, crossing-over, recombination rate, nucleolar organizer, NOR, bimodal karyotype, microchromosomes, heterochromatin, chiasma

## Abstract

**Simple Summary:**

Bimodal karyotypes, including both large chromosomes and microchromosomes, are mainly found in reptiles, birds, fish, and insects, but not mammals. Studies of microchromosomes are currently of great interest. The karyotype of the snake *Vipera berus* is a prime example of a bimodal karyotype. We conducted a comparative cytogenetic study of meiotic (synaptonemal complexes in prophase I) and mitotic chromosomes. A significant asynchrony in the assembly of meiotic bivalents and the dynamics of the appearance of the mismatch repair protein MLH1 were analyzed, and a high level of meiotic recombination was shown. Furthermore, minor species-specific markers of the *V. berus* meiotic karyotype were identified.

**Abstract:**

*Vipera berus* is the species with the largest range of snakes on Earth and one of the largest among reptiles in general. It is also the only snake species found in the Arctic Circle. *Vipera berus* is the most involved species of the genus *Vipera* in the process of interspecific hybridization in nature. The taxonomy of the genus *Vipera* is based on molecular markers and morphology and requires clarification using SC-karyotyping. This work is a detailed comparative study of the somatic and meiotic karyotypes of *V. berus,* with special attention to DNA and protein markers associated with synaptonemal complexes. The karyotype of *V. berus* is a remarkable example of a bimodal karyotype containing both 16 large macrochromosomes and 20 microchromosomes. We traced the stages of the asynchronous assembly of both types of bivalents. The number of crossing-over sites per pachytene nucleus, the localization of the nucleolar organizer, and the unique heterochromatin block on the autosomal bivalent 6—an important marker—were determined. Our results show that the average number of crossing-over sites per pachytene nucleus is 49.5, and the number of MLH1 sites per bivalent 1 reached 11, which is comparable to several species of agamas.

## 1. Introduction

The term “viper” is often used to refer to reptiles of the subfamily Viperinae. This subfamily includes 13 genera of snakes rather diverse in terms of morphological criteria that are widespread on all continents of the Old World except for Madagascar [1]. The topographic origin of vipers is still debated, but it certainly was not Europe [2,3,4]. Snakes of the genus *Vipera* (Laurenti, 1768) are the most common venomous snakes in Europe and Western and Central Asia [5]. The number of species varies from 10 to 20 according to different studies, since the taxonomic status of some species and subspecies remains questionable [6,7,8,9]. These snakes inhabit extremely diverse ranges—from deserts to alpine meadows and even northern territories beyond the Arctic Circle [10].

One of the youngest evolutionary forms is the genus *Vipera*, or True vipers [11]. Particularly rapid speciation of this genus took place during the Pliocene and Pleistocene [12,13].

The most widespread species among true vipers and snakes in general, is *Vipera berus* (Linnaeus, 1758). It can be found from west to east, from the British Isles to the Sakhalin Islands, and from north to south, from Scandinavia to the Balkans [14,15]. In addition, the common viper is the only snake found in the Arctic Circle [16,17].

Numerous studies describe the ecology and variety of forms of the genus *Vipera* [18,19,20,21,22,23,24,25,26,27,28,29,30,31,32], including proteomic studies of the characteristics of their venom [33,34,35,36] and medical studies of their antiproliferative and cytotoxic effects in oncosuppression [10,37,38].

On the other hand, there are only few comparative molecular biological studies of the species [39,40,41,42]. Cytogenetic studies of the *Vipera* genus were carried out mainly on mitotic metaphase chromosomes or the preparation of meiocytes using light microscopy [43,44,45,46,47,48].

The most interesting problems of natural [9,49,50,51,52,53] or laboratory [54] hybridization between different viper species have not yet been studied using molecular cytogenetic approaches. Moreover, there is still no detailed comparative analysis of somatic and meiotic karyotypes of the key species involved in hybridization.

Bimodal karyotypes, i.e., karyotypes with a significant difference in the sizes of macro- and microchromosomes, have been described in reptiles [55,56], birds [57,58,59], amphibians [60], fish [61,62], and some insects [63], but surprisingly not in mammals [64]. The question of why natural selection fixed these small chromosomes in each specific lineage remains unresolved. There is great interest in such karyotypes for several reasons: they raise the question of the independent evolution of macro- and microchromosomes [55], and they present data on low heterochromatin levels in microchromosomes, higher recombination rates, a higher-mutation rate, and higher gene density in microchromosomes [64,65]. Enrichment of specific genes in microchromosomes has been revealed in snakes [64,65,66,67]. Comparative studies in several snake taxa demonstrated the conservative structure of their macro- and microchromosome sets [67]. In general, snakes could have achieved genomes characterized by higher levels of compartmentalization and smaller chromosomes, possibly resulting in an increased frequency of recombination and a greater level of speciation [68].

Synaptonemal complex (SC) karyotyping is one of the most informative methods for studying molecular markers at the chromosomal level [69]. 

The aim of this work was a detailed comparative cytogenetic study of the somatic and meiotic karyotypes of the most widespread Viperidae species, *V. berus*, to identify minor species-specific characteristics of the SC-karyotype which are nondetectable on the mitotic chromosomes only, to study centromere positions in microchromosomes, nucleolar organizer location, and the distribution of the crossing-over marker, namely, the MLH1 protein. 

We believe that the pronounced bimodality of viper karyotypes can provide an excellent model for cytogenetic and genomic studies in Viperidae, snakes, and vertebrates as well.

## 2. Materials and Methods

### 2.1. Specimens

Three adult males and two females of *V. berus* were captured in 2019–2022 in the Tver region, Konakovsky district, and examined from May to October 2022. The manipulations with the animals followed the international rules of the Manual on Humane Use of Animals in Biomedical Research.

### 2.2. Mitotic Chromosome Preparation

Mitotic chromosome preparations were obtained from male and female individuals using a direct suspension technique described below. The bone marrow form ribs and the spleens were suspended in 10 mL of a 0.075 M KCl hypotonic solution and incubated for 20 min at room temperature; then, 1 mL of the freshly prepared 3:1 methanol–acetic acid fixative was added, and the cell suspension was centrifuged for 5 min at 1000 rpm. Afterwards, the supernatant was discarded, 5 mL of the fixative were added, and the cell suspension was kept at 4 °C for 15–20 min. These procedures were repeated two more times. After the third centrifugation and the elimination of the supernatant, 0.5–1.0 mL of the fixative was added, and the final cell suspension was left for storage at −20 °C. To prepare mitotic chromosome slides, several small drops of the cell suspension were released onto various sections of a slide previously maintained in distilled water at 4 °C; then, the slides were transferred to a hot plate (45 °C) for drying. The mitotic chromosome slides were stained conventionally with 4% Giemsa solution in a phosphate buffer solution at pH 6.8 for 8 min or mounted in a Vectashield antifade mounting medium with DAPI, 4′,6-diamidino-2-phenylindole (Vector Laboratories H-1200, Newark, CA, USA).

### 2.3. Total Preparation of SCs and Immunostaining

Seminiferous tubules were isolated and disaggregated in the phosphate-buffered saline (PBS) (PanEco, Moscow, Russia). A spread of spermatocytes I nuclei preparations were performed according to Spangenberg (2022) [69]. Poly-L-lysine-coated slides were used in all immunofluorescence studies. The slides stored in −20 °C were moved to room temperature, washed with phosphate-buffered saline (PBS) for 1 min, and incubated overnight at 4 °C with primary antibodies diluted in the antibody dilution buffer (ADB: 3% bovine serum albumin and 0.05% Triton X-100 in PBS). Axial elements of meiotic chromosomes were detected using rabbit polyclonal antibodies against the Synaptonemal complex protein 3 (SYCP3) protein (1:250; Abcam ab15093, Cambridge, UK). Centromeres were detected using the antikinetochore proteins’ antibodies ACA (1:500; Antibodies Incorporated 15–234, Davis, CA, USA), known also as CREST-syndrome antisera. Antibodies against DNA mismatch repair protein MLH1 (1:250; Abcam, Cambridge, UK) were used for the detection of the late recombination sites, prospective chiasmata. Nucleolus was detected by mouse antifibrillarin monoclonal antibodies (1:250; Abcam, Cambridge, UK). After washing in PBS, the secondary antibodies diluted in Antibody Dilution Buffer (ADB) were used, namely, goat antirabbit immunoglobulin G, Alexa Fluor 488 (1:500; Abcam, Cambridge, UK), goat antimouse Alexa Fluor 555 (1:500; Invitrogen, Carlsbad, CA, USA), and goat antihuman Alexa Fluor 555 (1:500; Invitrogen, Carlsbad, CA, USA). Incubation with secondary antibodies was performed in a humid chamber at 37 °C for 2 h.

### 2.4. Microscopy

The synaptonemal complex slides and mitotic chromosome slides, stained with DAPI, were examined using the Leica DM microscope equipped with the Axiocam HRm CCD camera and filter sets A, I3, and N2.1, and processed with AxioVision Release 4.8. software (Carl Zeiss, Oberkochen, Germany). All preparations were mounted in a Vectashield antifade mounting medium with DAPI (Vector Laboratories H-1200, Newark, CA, USA). The mitotic chromosome slides, conventionally stained with Giemsa, were examined using an Axioplan 2 Imaging microscope (Carl Zeiss, Germany) equipped with a CV-M4+CL camera (JAI, Kanagawa, Japan) and the Ikaros software (MetaSystems, Altlussheim, Germany).

### 2.5. Image Analysis

Synaptonemal complex measurements were performed with ImageJ software, release 1.53k (Bethesda, AR, USA). Criteria of identification of distinct meiotic prophase I stages were used in accordance with our previous studies in reptiles [70,71]. The Origin Pro software package (OriginLab Corp., Northampton, MA, USA) was used for descriptive statistics and diagram construction.

Mitotic and synaptonemal complex karyotypes were arranged according to the centromere position following Levan et al. (1964) [72] but modified as metacentric (m), sub-metacentric (sm), and sub-telocentric/acrocentric (st/a). Chromosome pairs were arranged according to their size. To determine the chromosomal arm number per karyotype (fundamental number, FN), metacentrics and sub-metacentrics were considered as biarmed and sub-telocentrics/acrocentrics as monoarmed.

## 3. Results

### 3.1. Mitotic Metaphase Karyotyping and Karyotypic Formula

**Figure 1:**
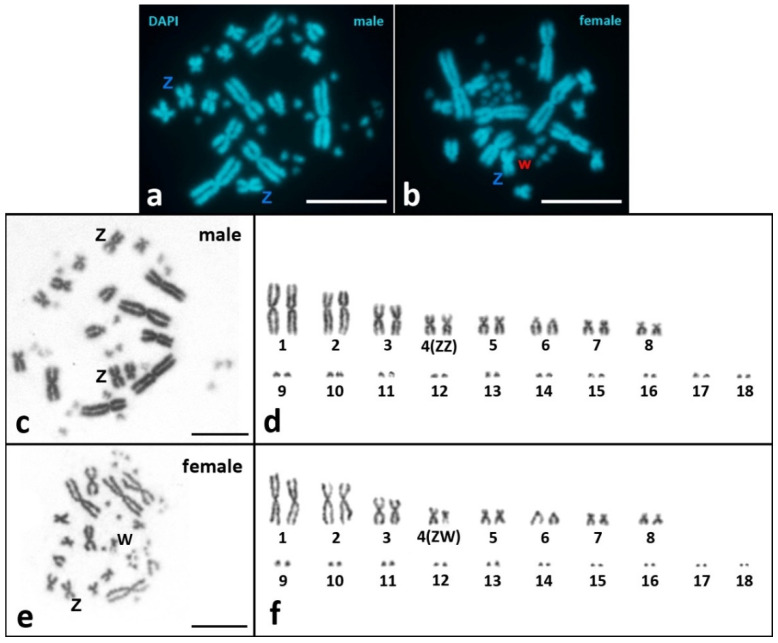
Mitotic karyotypes of *V. berus*. (**a**,**b**) DAPI staining and (**c**–**f**) conventional Giemsa staining. (**a**,**c**,**d**) Male karyotypes; (**b**,**e**,**f**) Female karyotypes. (**a**–**c**,**e**) Metaphase chromosome plates; (**d**,**f**) karyograms. ZZ/ZW—sex chromosomes. Scale bar—10 μm.

**Figure 2:**
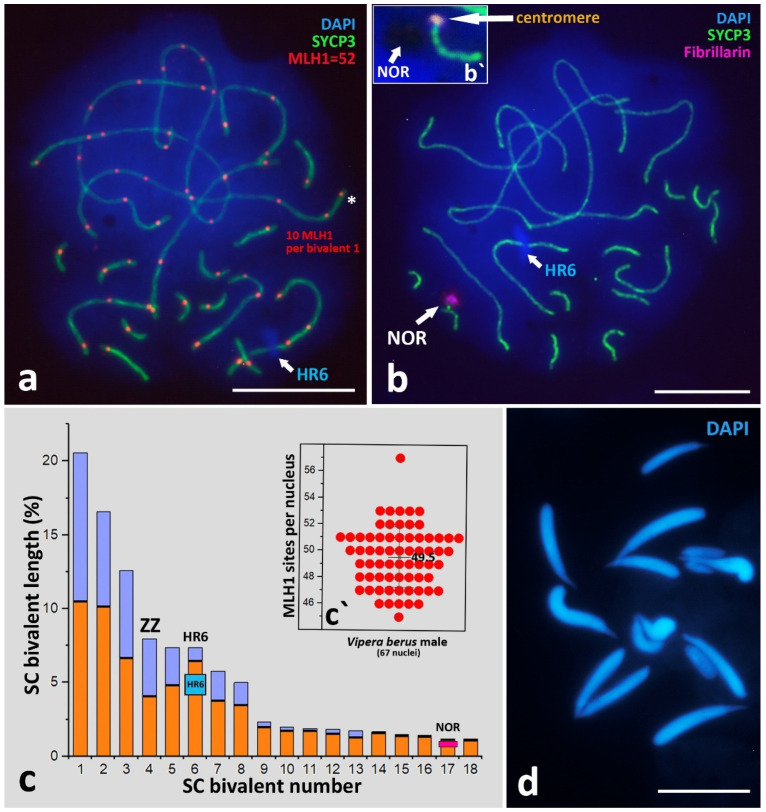
Markers of *V. berus* SC-karyotype, ideogram, and spermatids morphology. (**a**) Immunodetection of the crossing-over marker, MLH1 protein. A total of 52 MLH1 sites per nucleus, and 10 MLH1 sites on the bivalent 1 (asterisk). (**b**) Immunodetection of Nucleolar Organizer (NOR) on the microchromosome bivalent and (**b`**) as the DAPI-negative region. Axial elements of chromosomes are immunostained with antibodies against the SYCP3 protein (green); crossing-over sites are immunostained with antibodies against MLH1 protein (red); NOR with antibodies against Fibrillarin protein (violet). Chromatin stained with DAPI (blue). (**c**) Ideorgam of *V. berus* SC-karyotype. Blue—p arms; orange—q arms. Metacentric Z-chromosome, heterochromatin region (HR6), and NOR are indicated. Heterochromatin region on the chromosome pair 6 is indicated as HR6. (**c`**) Number of MLH1 foci per spermatocyte nucleus (mean ± SD). (**d**) Spermatids, DAPI staining (blue). Bar—10 μm.

Both male and female mitotic karyotypes of *V. berus* have 2n = 36 and consist of 6 metacentrics (pairs 1, 3, and 4), 8 sub-metacentrics (pairs 2, 5, 7, and 8), 2 subtelocentrics/acrocentrics (pair 6), and 20 microchromosomes (pairs 9–18) (Figure 1). Pair 4 is homomorphic in the male karyotype (ZZ) and heteromorphic in the female one (ZW) (Figure 1d,f). Z and W chromosomes are metacentric. The W chromosome is similar in size to pairs 7 and 8 but differs from them in morphology (pairs 7 and 8 are sub-metacentric) (Figure 1d). DAPI staining revealed the presence of an AT-poor region on the long arm of the W chromosome (Figure 1b). The morphology of the microchromosomes is not distinguishable after mitotic metaphase karyotyping. However, analysis of synaptonemal complexes (below in the Section 3.2.2.) using SYCP3 and ACA immunostaining reveal the morphology of microchromosomes: 2 microchromosomes are sub-metacentric (pair 13) and 18 microchromosomes are sub-telocentric/acrocentric (pairs 9–12 and 14–18) (Figure 2c). Thus, the karyotypic formula for both males and females of *V. berus* is 6m + 10sm + 20st/a, FN = 52.

### 3.2. Immunocytochemical Analysis of Meiotic Prophase I Nuclei of V. berus spermatocytes I

A total of 338 spermatocyte nuclei of *V. berus* at different stages of meiotic prophase I were studied. Immunostaining of protein markers allowed us to describe, in detail, all stages, which for convenience are divided into presynaptic, alignment, and postsynaptic stages.

#### 3.2.1. Presynaptic Stages

##### Leptotene

**Figure 3:**
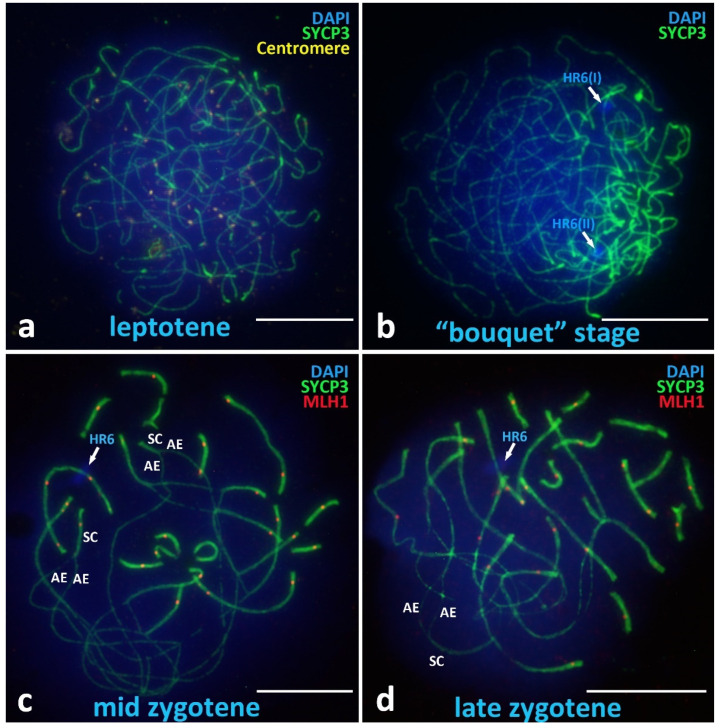
Presynaptic stages in *V. berus* primary spermatocytes. (**a**) Leptotene, long axial elements are completely asynapted, centromeres distributed over all spread nuclei. (**b**) Chromosomal ‘bouquet’ stage, U-shaped axial elements of chromosomes, clusterization of telomere ends in the local region of the spread nucleus. (**c**) Mid zygotene, asynchronous synapsis of the long chromosomes and microchromosomes. MLH1 protein loaded in bivalents of microchromosomes as well as in the regions of local synapsis of long assembling bivalents. (**d**) Late zygotene, finalization of assembly of the long bivalents. Bivalents of microchromosomes are fully assembled. Heterochromatin region on the chromosome pair 6 is indicated as HR6. AE—axial elements of asynapted chromosomes. SC—assembled regions of synaptonemal complexes. Axial elements of chromosomes are immunostained with antibodies against the SYCP3 protein (green), centromeres with antikinetochore antibodies ACA (yellow). Mismatch repair protein sites are immunostained with antibodies against MLH1 protein (red). Chromatin stained with DAPI (blue). Bar—10 μm.

The leptotene stage of *V. berus* primary spermatocytes has characteristics according to the classical description in the scientific literature as the “stage of thin long threads” or “tangled mass of threads” (Figure 3a) [73,74], which differs from those previously studied in reptiles, where the leptotene has mostly fragmented axial elements [70].

##### Chromosomal “Bouquet” Stage

The chromosomal “bouquet” stage demonstrates clustering of the telomeric ends of all univalents and U-shaped chromosomes (Figure 3b). At this stage, we have not been able to identify clear differences between macro- and microchromosomes.

##### Zygotene

The zygotene stage in *V. berus* is the most remarkable meiotic prophase I stage and is characterized by asynchronous assembly of synaptonemal complexes (Figure 3c,d). In the zygotene stage, differences in the dynamics of the assembly of both types of bivalents (macro- and micro-) are clearly visible. In the middle zygotene stage (Figure 3c), all the small bivalents are already assembled in synaptonemal complexes, while all the long bivalents demonstrate significant regions of asynaptic axial elements (AE) and only short peritelomeric regions of local synapsis (SC). In the late zygotene stage, only small areas of asynapsis remain in the interstitial areas of large bivalents (Figure 3d). It is important to note the fairly early loading of the MLH1 and the mismatch repair protein (prospective chiasma sites) into bivalents already at the zygotene stage. MLH1 protein sites are found both in fully assembled short bivalents as well as in regions of partial synapsis of the long bivalents (Figure 3c,d).

#### 3.2.2. Alignment Stage and Postsynaptic Stages

##### Alignment Stage

**Figure 4:**
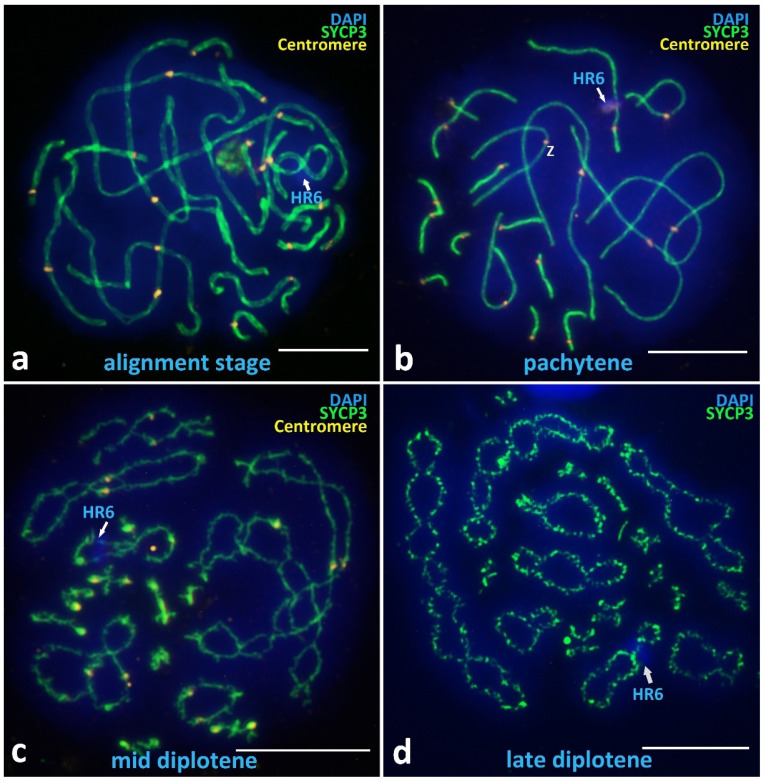
Alignment stage and postsynaptic stages in *V. berus* primary spermatocytes. (**a**) Alignment stage, spatial alignment of pairs of axial elements of homologous chromosomes located opposite each other. (**b**) Pachytene, complete synapsis of all 18 bivalents. (**c**) Mid diplotene, desynapsis of homologues, chiasmata. (**d**) Late diplotene, diffuse axial elements, unloading of the SYCP3, chiasmata. Heterochromatin region on the chromosome pair 6 is indicated as HR6. Axial elements of chromosomes are immunostained with antibodies against the SYCP3 protein (green), centromeres with antikinetochore antibodies ACA (yellow). Mismatch repair protein sites are immunostained with antibodies against MLH1 protein (red). Chromatin stained with DAPI (blue). Bar—10 μm.

Among the identified meiotic prophase I stages in *V. berus*, a rare stage of chromosome alignment should be noted, which was found in only 5 of the 338 nuclei studied (Figure 4a). This stage, which is described in many species, including reptiles [70], is characterized by the spatial alignment of pairs of axial elements of homologous chromosomes opposite to each other, separated by about two times as far as in the assembled SCs with clearly visible space between axes, and there is no loading of MLH1 yet [74].

##### Pachytene

The pachytene nuclei have complete synapsis of all 18 bivalents (Figure 4b and Figure A3), easily defined bivalent arm lengths separated by immunostained centromeres (Figure 4b), and fused NORs located on one of the microchromosome bivalents (Figure 2b). A specific problem in the majority of *V. berus* bimodal SC-spread preparations is the overlap and entanglement of the first three very long macrobivalents. To solve this problem, a large number of photographs were taken, and the most suitable were selected for measurements and analysis. Nevertheless, pachytene nuclei are most convenient for studying the basic markers of meiotic prophase I, which we describe in detail below in the section “SC-karyotyping and meiotic prophase I markers analysis”.

##### Diplotene

The diplotene stage is characterized by desynapsis of homologues and gradual unloading of the SYCP3 protein from axial (lateral) elements (Figure 4c). In the late diplotene, the chromosome axes become diffuse (Figure 4d). At the diplotene stage, chiasmata are clearly visible, the number of which corresponds to the number of MLH1 sites in pachytene, including those for macrobivalents (Figure 2a and Figure 4c,d).

#### 3.2.3. SC-Karyotyping and Meiotic Prophase I Markers Analysis

##### Crossing-Over Marker, MLH1 Protein

Immunostaining of crossing-over associated protein MLH1 revealed a surprisingly high number of sites per pachytene nucleus. The findings revealed an average of 49.5 ± 2.27 (mean ± SD) MLH1 sites (a minimum of 45 sites and a maximum of 57) on 18 pachytene bivalents (n = 18, FN = 52) (Figure 2a and Figure A3a). In addition, *V. berus* pachytene nuclei demonstrated a high number of MLH1 sites per one macrochromosome bivalent. Here, we revealed up to 11 MLH1 foci on the bivalent (Figure A3b). Ten microchromosomes have, on average, 11.06 ± 1.01 MLH1 sites.

##### Unique Heterochromatic Chromatin Region (HR6) on the Bivalent 6

**Figure A1:**
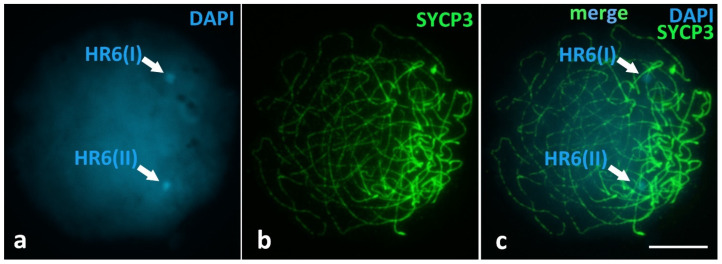
Chromosomal “bouquet” stage, *V. berus* spermatocyte I. Detection of the heterochromatin regions on the both chromosomes 6 before synapsis (HR6(I) and HR6(II)). (**a**) Chromatin stained with DAPI (blue), (**b**) Axial elements of chromosomes are immunostained with antibodies against the SYCP3 protein (green), and (**c**) merge. Bar—10 μm.

**Figure A2:**
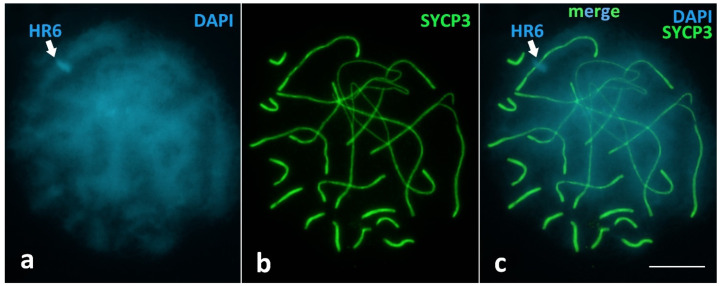
Late zygotene stage, *V. berus* spermatocyte I. Heterochromatin region on the bivalent 6 (HR6) after the synapsis of two homologous chromosomes 6. (**a**) Chromatin stained with DAPI (blue), (**b**) Axial elements of chromosomes are immunostained with antibodies against the SYCP3 protein (green), and (**c**) merge. Bar—10 μm.

**Figure A3:**
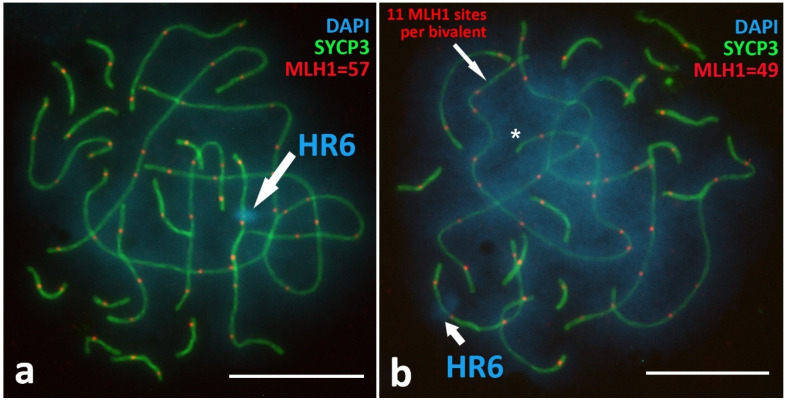
Pachytene stage, *V. berus* spermatocyte I. Immunodetection of crossing-over marker, MLH1 protein (red). (**a**) 57 MLH1 sites in one SC-spread. (**b**) 49 MLH1 sites in one SC-spread and 11 MLH1 sites on one macrobivalent 1 (asterisk). Heterochromatin region on the bivalent 6 (HR6) is indicated. Axial elements of chromosomes are immunostained with antibodies against the SYCP3 protein (green). Chromatin stained with DAPI (blue). Bar—10 μm.

One important finding is the heterochromatin region that was clearly visible on SC-spreads on the q arm of the bivalent 6 in all postzygotene nuclei we studied (Figure 3c,d and Figure 4a,d; Figure A2 and Figure A3), indicated as HR6. Bivalent 6 was assigned to the sub-telocentric/acrocentric (st/a) type. HR6 is a DAPI-intensive region, located separately from centromere but also detectable with immunostaining using ACA antibodies (Figure 4b). In addition, we confirmed the presence of HR6 before synapsis at the chromosomal “bouquet” stage as two separated heterochromatin blocks, HR6(I) and HR6(II), located separately on the yet asynapted axial elements (Figure 3b and Figure A1).

##### Sex Z Chromosome Identification in the SC-Karyotype

The immunostaining of centromere proteins on the pachytene bivalents (Figure 4b) and the data of mitotic karyotyping (Figure 1) allowed us to determine Z bivalent as the only metacentric bivalent 4, which was clearly different from the bivalent 5 (sm) and bivalent 6 (st/a) of similar length. Detailed data are presented in the ideogram (Figure 2c).

##### Nucleolar Organizer Region (NOR)

We detected NOR location using a combination of two criteria: immunostaining with antibodies against Fibrillarin protein and the “empty” region on the DAPI staining of chromatin (NOR is an RNA- and protein-enriched region which has very weak DAPI staining). Thus, we concluded that NOR is most likely located on the micro-bivalent 17 in the proximal region to the centromere (Figure 2b,b`). However, the difference between chromosomes 17 and 18 is so small that it does not allow us to detect accurately the localization of NORs.

##### Spermatids

Spermatids were observed on the SC preparations using fluorescent microscopy. Spermatids of *V. berus* displayed typical characteristics of reptiles, including an elongated head and uniform chromatin staining, which corresponded to descriptions of reptile sperms in other papers [75].

## 4. Discussion

In many species, tiny microchromosomes are morphologically indistinguishable (dot-shaped) on mitotic metaphase plates [63]. Molecular cytogenetic techniques such as whole chromosome painting and comparative genomic hybridization are powerful methods for comparing and identifying specific microchromosomes of interest in the preparation of mitotic metaphase plates [76,77]. Basic karyological information is crucial to link cytogenetic and genomic data [78,79,80].

On another hand, our results indicate the applicability of comparative studies of both the somatic and meiotic chromosomes for the most complete analysis of not previously described DNA and protein markers of the bimodal karyotype.

- Our results in mitotic chromosome karyotyping are in agreement with cytogenetic data obtained previously for *V. berus*, except for minor differences in the classifications of some macrochromosomes [44]. The karyotype of *V. berus* described by us consists of 16 macrochromosomes (6m + 8sm + 2st/a) and 20 microchromosomes (2sm + 18st/a), FN = 52. For the first time, we revealed the morphology of microchromosomes in *Vipera* using high-resolution SC-karyotyping. Karyotypes with 16 macro- and 20 microchromosomes were also described previously in *V. ursinii*, *V. latastei,* and *V. seoanei* [45,81]. On the other hand, *V. aspis* and *V. ammodytes* karyotypes with 22 macro- and 20 microchromosomes were reported [45,48,81]. The presence of two variants of karyotypes in the genus *Vipera* is intriguing, and further studies on both mitotic and meiotic chromosomes are needed to understand the evolution of the karyotypes in the genus.
- Preparations of metaphase plates obtained during the study showed a structured distribution of macro- and microchromosomes: microchromosomes clustered closer to each other, forming a “microchromosome zone”. This was most clearly observed on the weakly and medium-spread mitotic metaphase plates (Figure 1b,e). This partially corroborates the data of other authors, who describe a certain order of arrangement of chromosomes on metaphase plates [67]. Indeed, studies of bimodal karyotypes of birds and reptiles suggest that microchromosomes interact strongly and regularly locate together in somatic nuclei at interphase and during cell division, suggesting their functional coherence [67].
- Synaptonemal complex spread preparations provide detailed visualization of meiotic SC bivalents, which are from three- to five-times longer than mitotic metaphase chromosomes [82]. The *V. berus* karyotype is a striking example of a bimodal karyotype, combining both very large chromosomes and many microchromosomes. Thus, SC-karyotyping is a logical and very useful method in this regard, as it has allowed us to compare lengths, centromere and NOR positions, and the number of crossing-over sites in microchromosomes.

The performed immunocytochemical analysis of the stages of prophase I of meiosis makes it possible to analyze the dynamics of the synapsis of homologous pairs of macro- and microchromosomes. 

It should be noted that it was almost impossible to find the pachytene stage (with completely assembled SCs) on some slides of spermatocytes I spreads. On the contrary, partial asynapsis was always found in macrobivalents. This is due to the asynchronous assembly of bivalents: microchromosomes are far ahead of long chromosomes. Therefore, an important methodological result for us was the obligatory use of tissues from different sectors of the gonad. This method, in our opinion, can be recommended for working with preparations of synaptonemal complexes of organisms with bimodal karyotypes.

The number of crossing-over sites is often considered in the context of rates of evolution [78]. Here, we immunostained MLH1 sites in the bimodal pachytene SC-karyotype of *V. berus* (Figure 2a and Figure A3). The average number of MLH1 sites was 49.5 ± 2.27 (57 sites at maximum (Figure A3a)) on 18 pachytene bivalents (n = 18, FN = 52), which is a little lower than the crossing-over champion described in Agamidae with 69.2 MLH1 sites on 23 acrocentric bivalents (n = 23; FN = 46) [83]. Theoretically, at least one MLH1 site is needed on every bivalent for successful chiasmata formation and correct segregation in metaphase I to avoid aneuploidy of gametes. In *V. berus,* we detected an average of 11.06 ± 1.01 MLH1 sites on ten micro-bivalents, which is approximately 22.3% of the total number of MLH1 sites per nucleus (49.5 ± 2.27) (Figure 2a,c`). Of all the 18 SC bivalents in *V. berus*, these ten micro-bivalents take only 16.7% of the total SC length. Studies of bimodal karyotypes in birds and reptiles suggest that such chromosomal architecture may be connected with unequal rates of sequence evolution within one genome [64,84]. For instance, the enrichment of specific genes in microchromosomes and their intense evolution have been proposed for snakes [64,65]. On the other hand, seasonal variations in the frequency and distribution of chiasmata (terminal/intermediate) may be associated with the concentration of steroid hormones during a year [85].

The heterochromatic region we detected is a strong and easily detected marker revealed in all the nuclei under study. Our preliminary data (not shown) suggest this region is species-specific for *V. berus* and important for further comparative cytogenetic studies of closely related species of the genus *Vipera*. The fact that HR6 is detectable on anticentromere proteins immunostaining and is located separately from the centromere can be connected with the very broad specificity of these antibodies. Indeed, ACA (or CREST) antiserum is a cocktail of different anti-CENP protein family antibodies. Further studies are needed to detail the origin and specifics of the HR6 region in *V. berus* and other closely related species.

Localization of NOR on the pair of microchromosomes we revealed using antifibrillarin antibodies and DAPI staining is similar to our previous study of NOR in Lacertidae oocytes [86]. Our result is in accordance with known data on other snakes [48,87]. On the other hand, studies of several snake species using silver nitrate staining methods (AgNOR) or FISH revealed the location of NORs in two chromosome pairs [87,88,89]. In general, the *V. berus* karyotype shows several primitive characteristics, 2n = 36 with 16 macro- and 20 microchromosomes, fourth pair ZW heteromorphic sex chromosomes, and NORs on one microchromoosme pair described for snakes [90,91].

Further studies of Viperidae genomes are needed, such as the analysis of repetitive DNAs (rDNA, satellite DNA) and their distribution between macro- and microchromosomes [92]. Comparative SC-karyotyping of closely related species within the genus *Vipera* could help to determine the taxonomic status of many known forms and subspecies.

## 5. Conclusions

We performed a detailed comparative study of mitotic and meiotic karyotypes of *V. berus.* We described important protein and DNA markers, some of which are impossible to detail with mitotic metaphase karyotyping only. These markers allowed us to distinguish autosomal chromosomes of similar length, the sex chromosomes pair, and the localization of nucleolar organizer. 

We traced synaptonemal complexes assembly and disassembly on the successive stages of meiotic prophase I in the bimodal karyotype of *V. berus* and visualized the highly asynchronous synapsis of macro- and microchromosomes in detail.

We revealed that the average number of sites of the crossing-over marker MLH1 in *V. berus* is 49.5 per spermatocyte’s nucleus and up to 11 MLH1 sites on the largest bivalent 1. Furthermore, we detected up to 57 MLH1 sites, which is a very high rate and can be compared to the champion species from Agamidae.

The heterochromatin region HR6 that was detailed on the bivalent 6 is an important DNA marker of the *V. berus* karyotype, and it will be useful in future comparative studies.

In general, SC-karyotyping demonstrates high applicability and will complement future studies using chromosome-scale assemblies of bimodal karyotypes and genomic studies in general [56,78,79].

## Data Availability

Not applicable.
